# Efficacy of epidermal growth factor receptor-targeted molecular therapy in anaplastic thyroid cancer cell lines

**DOI:** 10.1038/sj.bjc.6602461

**Published:** 2005-03-22

**Authors:** Y Nobuhara, N Onoda, Y Yamashita, M Yamasaki, K Ogisawa, T Takashima, T Ishikawa, K Hirakawa

**Affiliations:** 1 Department of Surgical Oncology, Osaka City University Graduate School of Medicine, 1-4-3, Asahi-machi, Abeno-ku, Osaka 545-8585, Japan; 2 Department of Oncology, Institute of Geriatrics and Medical Science, Osaka City University Graduate School of Medicine, 1-4-3, Asahi-machi, Abeno-ku, Osaka 545-8585, Japan

**Keywords:** undifferentiated thyroid cancer, epidermal growth factor receptor, gefitinib

## Abstract

Anaplastic thyroid cancer is one of the most aggressive human malignancies and the outcomes of conventional therapy have been far from satisfactory. Recently, epidermal growth factor receptor (EGFR)-targeted therapy has been introduced as an alternative therapeutic strategy for highly malignant cancers. This study was undertaken to investigate the expression of EGFR in anaplastic thyroid cancer cell lines, and to explore the potential of therapies targeting EGFR as a new therapeutic approach. EGFR was universally expressed in anaplastic cancer cell lines at a variety of levels. Specific EGFR stimulation with epidermal growth factor showed significant phosphorylation of ERK1/2 and Akt, and resulted in marked growth stimulation in an anaplastic thyroid cancer cell line, which highly expressed EGFR. This EGFR-transmitted proliferation effect of the cancer cell line was completely inhibited by gefitinib, an EGFR tyrosine kinase inhibitor. Moreover, growth of xenografts inoculated in mice was inhibited in a dose-dependent manner with 25–50 mg kg^−1^ of gefitinib administrated orally. Inhibition of EGFR-transmitted growth stimulation by gefitinib was clearly observed in anaplastic thyroid cancer cell lines. Our results suggested that EGFR-targeted therapy, such as gefitinib, might be worth further investigation for the treatment of anaplastic thyroid cancer.

Anaplastic thyroid carcinoma, one of the most aggressive human malignancies, shows rapid invasive growth to the surrounding tissues and often demonstrates metastatic disease in the distant organs. In such cases, surgery alone is not enough and a combination therapy with chemotherapy and radiation is essential, yet the outcome of such intensive multimodal therapy is far from satisfactory. The majority of patients with anaplastic thyroid cancer die within a year after receiving an initial diagnosis (Volante *et al*, 2004). Different therapeutic approaches have been explored but the results are disappointing, and the prognosis has not changed over the past decades. Thus, a novel therapeutic approach is highly desirable.

The epidermal growth factor receptor (EGFR) is a cell membrane receptor that plays a key role in cancer development and progression. Increasing evidence has shown that the overexpression of EGFR closely correlates with advanced tumour stage and metastasis, and poor clinical outcome in many human cancers including breast, cervix, lung, bladder, and head and neck (Iihara *et al*, 1993; Hu *et al*, 1997; Grandis *et al*, 1998; Brabender *et al*, 2001; Arteaga and Truica, 2004). An EGFR can be stimulated upon interaction with corresponding ligands such as epidermal growth factor (EGF) or transforming growth factor-*α* (Ullrich and Schlessinger, 1990). Ligand binding to EGFR induces dimerisation of the receptor. Homo and/or heterodimerisation of EGFR activates intrinsic tyrosine kinase, leading to receptor autophosphorylation, then activates a number of intracellular signal transducing elements (Raymond *et al*, 2000). Phosphatidylinositol-3′-kinase, protein kinase B/AKT (Akt), a small G-protein (ras) the ras GTPase-activating protein, extracellular signal-regulated kinase (ERK) 1/2, Src family kinase, and STATs mediated pathway are the known downstream effecters of the EGFR. A cell-proliferating signal from the activated EGFR reaches ERK1/2 through ras/raf activation, and is transmitted to intranucleus cell proliferation signals. Akt also transmits the signal from activated EGFR to inhibit apoptosis.

Recently, molecular-targeted therapy has attracted attention. Many kinds of EGFR-targeted molecular treatments have been attempted, such as antireceptor monoclonal antibodies, antiligand monoclonal antibodies, ligand–toxin conjugates, scFv–toxin conjugates, ligand–genistein conjugates and tyrosine kinase inhibitors (Raymond *et al*, 2000). Among these, gefitinib (‘Iressa’, ZD1839) is an orally active EGFR tyrosine kinase inhibitor, which blocks EGFR signal transduction pathways. Antitumour activity of gefitinib has been shown in experimental animal models (Sirotnak, 2003) and tumour cell lines (Campiglio *et al*, 2004). Gefitinib has demonstrated significant antitumour activity in two phase II trials in advanced non-small-cell lung cancer (NSCLC), and is now approved for this indication in many markets around the world (Fukuoka *et al*, 2003; Kris *et al*, 2003).

In this study, we demonstrated the expression of EGFR in anaplastic thyroid cancer cell lines and explored the potential therapeutic benefits of targeting this molecule with gefitinib.

## MATERIALS AND METHODS

### Chemicals

Gefitinib (Iressa, ZD1839) was provided by AstraZeneca (Macclesfield, UK).

### Cell lines and cell cultures

We used five human undifferentiated thyroid cancer cell lines (OCUT-1, -2, TTA-1, KTC-1 and ACT-1). OCUT-1 (Ogisawa *et al*, 2002) and -2 were recently established and characterised in our laboratory. OCUT-2 was established from cancerous fluid in the thoracic cavity of an 81-year-old Japanese woman with anaplastic thyroid cancer. This cell line was passed over 100 passages, grown stably and maintained over 2 years from the initial primary culture. TTA-1, KTC-1 and ACT-1 were each kindly provided by Dr A Yoshida of Kanagawa Cancer Center, Dr J Kurebayashi of Kawasaki Medical College and Dr S Ohata of Tokushima University, respectively. Each cell line was cultured in Dulbecco’s modified Eagle’s medium (DMEM) supplemented with 10% fetal bovine serum (FBS), 100 IU ml^−1^ of penicillin and 100 *μ*g ml^−1^ of streptomycin at 37°C with 5% CO_2_ in a humidified condition.

### Reverse transcription–polymerase chain reaction

We determined the expression of EGFR mRNA by reverse transcription–polymerase chain reaction (RT–PCR) as reported previously (Ogisawa *et al*, 2002). Briefly, total RNA was collected from samples using Trizol reagent (Life Technologies, Inc., Gaithersburg, MD, USA). Total RNA (1 ng) was reverse-transcripted in 20 *μ*l of reaction buffer containing 1 *μ*l of oligo dT primer, 4 *μ*l of 5 × RNA PCR buffer, 1 *μ*l of 10 mM dNTPs, 2 *μ*l of 0.1 M DTT, 0.5 *μ*l of RNA guard (Amersham Pharmacia Biotech, Buckinghamshire, UK) and 1 *μ*l of Moloney murine leukemia virus reverse transcriptase (Life Technologies). cDNA samples were amplified in 20 *μ*l of PCR reaction mixture with each primer set and *Taq*-polymerase (AmpliTaq Gold, Applied Biosystems, Tokyo, Japan). The primers used for EGFR were 5′-CGC TGC TGG CTG CGC TCT G-3′, 5′-CCT CCT GGA TGG TCT TTA T-3′ (Ullrich *et al*, 1984). Reverse transcription–polymerase chain reaction efficiency was confirmed by amplifying human glyceraldehydes-3-phosphate dehydrogenase (GAPDH), using the primers 5′-ACC ACA GTC ATG CCA TCA C-3′ and 5′-TCC ACC ACC CTG TTG CTG TA-3′. PCR conditions were 94°C for 3 min, followed by 30 cycles of 94°C for 30 s, 65°C for 30 s and 72°C for 30 s. Amplified products were electrophoresed on 2% agarose gel, stained with ethidium bromide.

### Flow cytometry

Expression of EGFR on the cell surface was measured by flow cytometry (Zhang *et al*, 2004). Five cell lines were plated in 100 mm dishes. Cells were left for 48 h to grow to semiconfluency then lifted with trypsin treatment and aliquoted into two sets of tubes (1 × 10^6^ cells/tubes), and washed twice with cold FACS buffer (0.03% sodium azide, 0.3% bovine serum albumin in phosphate-buffered saline (PBS)). One set of cells was treated with 200 *μ*g ml^−1^ of EGFR monoclonal antibody (F0797 DAKO). Cells not treated with monoclonal antibody were used as a control. All incubations were performed for 30 min in the dark on ice. Samples were again washed with cold FACS buffer, and incubated with 2 *μ*g of fluorescein isothiocyanate (FITC)-conjugated goat antimouse IgG (Chemicon Int. Inc., Temecula, CA, USA) in 200 *μ*l of FACS buffer for 30 min on ice. After washing again with FACS buffer, FITC-labelled cells were scanned using a FACS/Calibur Flow Cytometer (Becton Dickinson, Mountain View, CA, USA).

### MTT assay

The inhibitory effects of gefitinib on the viability of these cell lines were measured by MTT assay (Chung *et al*, 2002). Cells (1 × 10^4^) were seeded in each well of a 96-well plastic culture plate and left overnight under the same conditions. They were then treated with the intended doses of gefitinib for 3 days. After the incubation period, MTT was added to the final concentration of 0.5 mg ml^−1^, and the cells were incubated again for 2 h under the same conditions. The culture plate was centrifuged at 200 × **g** for 5 min and the supernatant was removed. Dimethyl sulphoxide was added for reaction, and the absorbency was measured with a microplate reader (Model 550, Bio-Rad Laboratories, Hercues, CA, USA) and calculated using the supplied software. The experiments were carried out three times independently, in triplicate each time, and the average values of the three independent experiments were calculated.

### Effect of gefitinib on tumour cell proliferation under EGF stimulation *in vitro*

Approximately 4 × 10^4^ cells were spread onto a 10-mm plastic dish and left overnight. Then the cells were cultured for 48 h in DMEM without FBS. In total, 1 nmol of EGF (#26190U, Upstate, Lake Placid, NY, USA) was added to a plate to stimulate the EGFR of the cells. The efficacy of gefitinib (10 and 100 nM) or neutralising antibody (#26190U, Upstate) was investigated by adding them to EGF. The number of cells was counted after 48 h of incubation. Experiments were carried out independently in triplicate.

### Western blotting

Expression of the protein in the signal transduction pathway was measured by Western blot analysis (Ono *et al*, 2004). Cells were incubated for 48 h supplemented with EGF (1 nM), or EGF (1 nM) and gefitinib (10 or 100 nM) as explained above. After the treatment, cells (1 × 10^6^) were lysed in 200 *μ*l of 1% triton X in PBS and gently shaken for 20 min, the protein was then extracted by centrifugation at 8050 × **g** for 10 min at 4°C. Protein concentration was measured and total protein (60 *μ*g) was electrophoresed on a 10% polyacrylamide gel and transferred onto a PVDF membrane (Hybond P, Amersham Pharmacia Biotech). The membrane was blocked with 5% skim milk for 2 h at room temperature, and incubated with 1 : 1000 dilution of p44/42 MAP kinase and phospho-p44/42 MAP kinase rabbit antibody (# 9101, 9102 Cell Signaling technology) or 1 : 1000 dilution of Akt and phospho-Akt rabbit antibody (# 9271, 9272 Cell Signaling Technology) for 12 h at 4°C. After three washings with 0.1% Tween 20 in PBS for 10 min each at room temperature, the membrane was incubated for 1 h at room temperature with peroxidase-conjugated secondary antibody (AP181P, Chemicon International, Inc., Temecula, CA, USA), and again washed five times with PBS under the same conditions. Peroxidase activity of the secondary antibody was detected with an enhanced chemi-luminescence detection system (ECL Plus Western Blotting Detection System, Amersham Pharmacia Biotech).

### *In vivo* experiment to confirm the efficacy of gefitinib in xenografts of cancer cells

Female balb/ca Jcl-nu mice, 4 weeks old, was purchased from Japanese Kurea Co., Osaka, Japan. The ethical issues of the experiment were approved by the Animal Research Committee of Osaka City University Graduate School of Medicine, and the animals were maintained in accordance with institutional guidelines. Mice were acclimatised at the Animal Facility of Osaka City University Graduate School of Medicine for 1 week. Then, mice were injected subcutaneously with 10^7^ ACT-1 or OCUT-2 cells into the dorsal flank under anaesthesia. After 7 days, when established tumours of approximately 3–4 mm in diameter were detected, mice were assigned to one of three treatment groups (*n*=8 in each group). Each group of mice were administered with 0, 25 or 50 mg kg^−1^ of gefitinib p.o. once a day on days 1–5 each week for 4 weeks as a ball-milled suspension in 0.5% (v v^−1^) polysolvate 80 (Wakeling *et al*, 2002). The mice were monitored daily for signs of toxicity and were weighed regularly. Tumour size was measured using the formula *π*/6 × larger diameter × (smaller diameter)^2^, as reported previously (Ciardiello *et al*, 2000).

### Immunohistochemistry

Immunoreactivity for EGFR and CD34 was determined by the streptoavidin–biotin method. Formalin-fixed, paraffin-embedded tissue blocks were obtained by xenograft. An immunohistochemical study was performed as described previously (Ogisawa *et al*, 2002). Briefly, sections were dewaxed, microwave pretreated and incubated with 0.3% hydrogen peroxide in methanol for 30 min. After blocking to reduce nonspecific antibody binding, polyclonal rabbit anti-human EGFR antibody (Santa Cruze Biotechnology, Santa Cruze, CA, USA) (Hirata *et al*, 2004) and monoclonal antibody against CD34 (DAKO M7168) were reacted with tissue sections at room temperature for 2 h followed by three washes with PBS. The sections were incubated with secondary antibody, and then reacted with streptoavidin–biotin peroxidase regent (HISTOFINE KIT, Nichirei Co., Tokyo, Japan). Finally, diaminobenzidine and 1% hydrogen peroxidase were applied as chromogen, counterstained with haematoxyline.

## RESULTS

### Expression of EGFR and effects of gefitinib on cell viability in cancer cells

**Figure 1:**
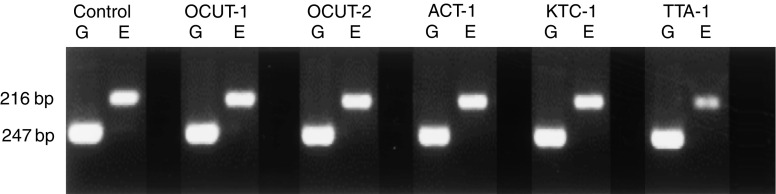
Expression of EGFR mRNA was determined by RT–PCR. A clear EGFR mRNA expression was observed in every cell line examined. ‘G’ and ‘E’ represented GAPDH and EGFR transcript, respectively. Oesophageal cancer cell line TE-1 was used for positive control.

**Figure 2:**
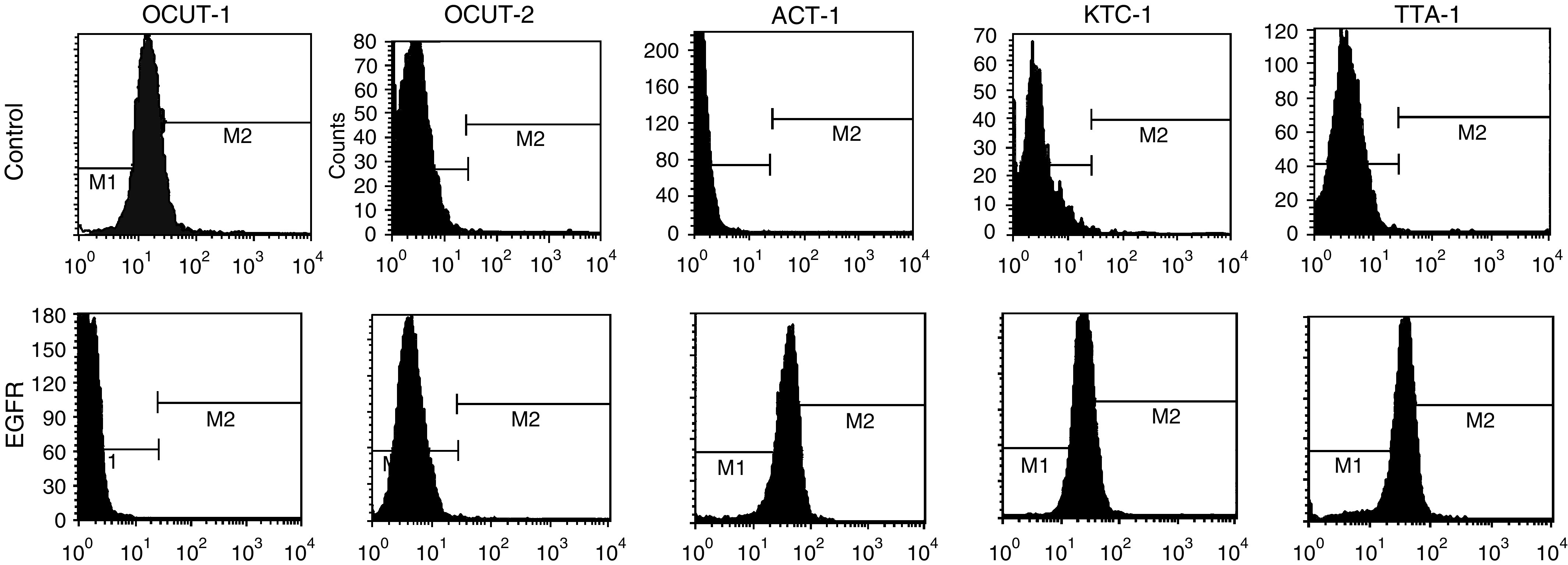
Expression of EGFR on the cellular surface was determined by flow cytometric analysis. The levels of EGFR expression in each cell lines varied. Expression level was the lowest in OCUT-2 compared with other cell lines. High-level expression of EGFR was shown in ACT-1, KTC-1 and TTA-1 cell line. There was no correlation between the level of mRNA and protein expression.

**Figure 3:**
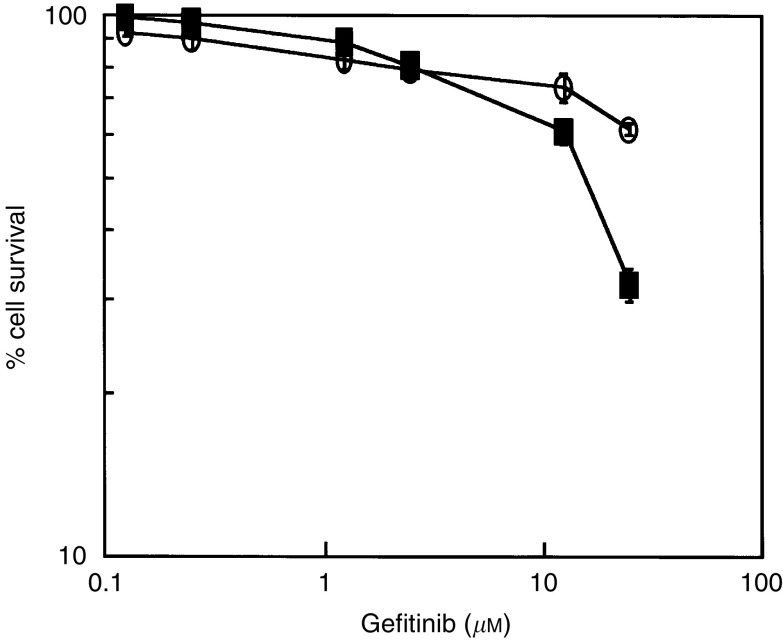
Efficacy of gefitinib on cell viability was determined by MTT methodology. Inhibitory effects of gefitinib were observed in every cell line examined under normal culture conditions with 10% FBS. The inhibitory effect was most significantly found in the ACT-1 cell line (closed square), which overexpresses EGFR, than in the OCUT-2 cell line (open circle), which expresses the lowest level of EGFR of all the cell lines. High concentrations of gefitinib (>10 *μ*M) were required to display a significant adverse effect on cell viability in the presence of FBS.

Expression of EGFR mRNA and protein was observed in every cell line examined (Figure 1). The level of EGFR expression varied and was lowest in OCUT-2 compared with other cell lines. High-level expression of EGFR was shown in both ACT-1 and TTA-1 cell lines (Figure 2). There was no correlation between the level of mRNA and protein expression. Dose-dependent effects of gefitinib on cell viability were observed in every cell line examined under normal culture conditions with 10% FBS. The inhibitory effect of gefitinib on cell viability was more prominent in the ACT-1 cell line, which overexpressed EGFR, than the OCUT-2 cell line, which expressed low levels of EGFR (Figure 3). However, even for the EGFR-overexpressing cell line, ACT-1, high concentrations of gefitinib (>10 *μ*M) were required to display a significant effect on cell viability in the presence of FBS. We could not maintain the cells without existence of FBS for more than 72 h, and could not get any reliable data from the similar MTT assays without FBS. Thus, we investigated the effect of gefitinib on EGF stimulation.

### Effect of gefitinib on tumour cell proliferation under EGF stimulation

**Figure 4:**
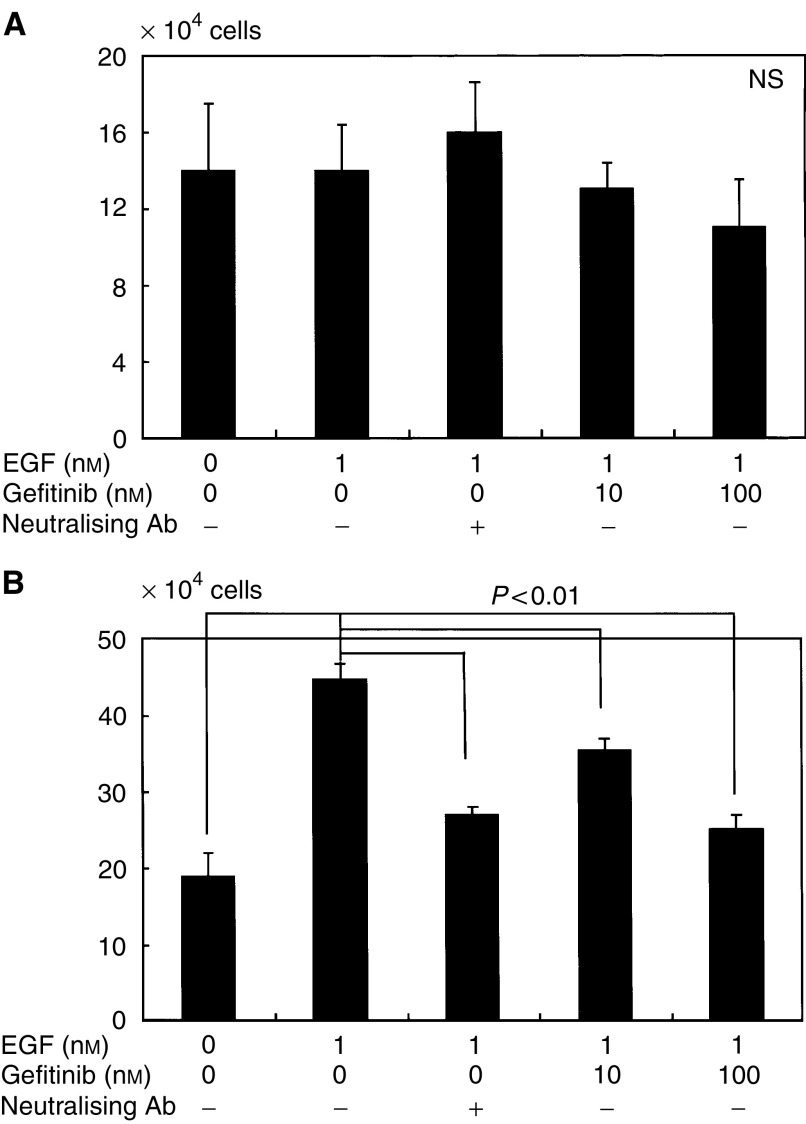
The effect of gefitinib on cancer cell proliferation under EGF stimulation was investigated in two cell lines (OCUT-2 and ACT-1). The number of cells was counted after 48 h following EGF stimulation. Although no stimulatory effect of EGF was displayed in OCUT-2 (**A**), which faintly expresses EGFR, a marked stimulatory effect of EGF on cell proliferation was displayed in the ACT-1 cell line (**B**), which overexpresses EGFR. A significant inhibitory effect of gefitinib on growth stimulation by EGF was recognised in a dose-dependent manner in ACT-1. The dose of gefitinib required to inhibit the effect of EGF stimulation was as low as 100 nM, and was as effective as EGF neutralisation.

The effect of gefitinib on cancer cell proliferation under EGF stimulation was investigated in two cell lines (OCUT-2 and ACT-1). No stimulatory effect of EGF was displayed in OCUT-2, which faintly expressed EGFR (Figure 4A). No significant inhibition of gefitinib upon cell growth was observed in OCUT-2. In contrast, a marked stimulatory effect of EGF on cell proliferation was displayed in the ACT-1 cell line, which overexpressed EGFR (Figure 4B). An inhibitory effect of gefitinib on ACT-1 cell growth stimulated by EGF was recognised in a dose-dependent manner. The dose of gefitinib required to inhibit the effect of EGF stimulation was as low as 100 nM and was as effective as EGF neutralisation. Moreover, gefitinib itself did not inhibit ACT-1 cell growth in the absence of EGF, suggesting that the growth inhibitory effect of gefitinib was mainly presented through inhibition of the EGFR-mediated cell-proliferative pathway.

### Effect of gefitinib on phosphorylation of ERK1/2 and Akt

**Figure 5:**
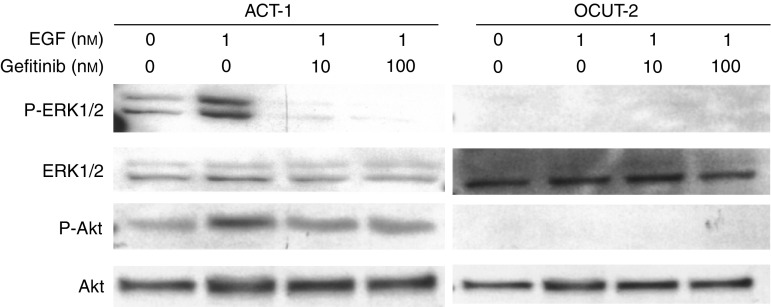
Effect of gefitinib on phosphorylation of ERK1/2 and Akt was shown by Western blot analysis. EGF-induced phosphorylation of ERK1/2 and Akt was clearly observed in the ACT-1 cell line. The inhibitory effect of gefitinib on EGF-induced phosphorylation of ERK1/2 was significantly observed in the ACT-1 cell line. The phosphorylation of ERK1/2 was almost completely inhibited in this cell line. The phosphorylation of Akt was also inhibited by gefitinib in the ACT-1 cell line overexpressing EGFR.

Figure 5 shows the result of Western blot analysis. EGF-induced phosphorylation of ERK1/2 and Akt was clearly observed in the ACT-1 cell line. However, in the OCUT-2 cell line, no evident phosphorylation of ERK1/2 and Akt was observed by EGF stimulation. The inhibitory effect of gefitinib on EGF-induced phosphorylation of ERK1/2 was significantly observed in the ACT-1 cell line. The phosphorylation of ERK1/2 was almost completely inhibited in this cell line. The phosphorylation of Akt was also inhibited by gefitinib in the ACT-1 cell line overexpressing EGFR.

### Effect of gefitinib on the growth of xenografts in nude mice

**Figure 6:**
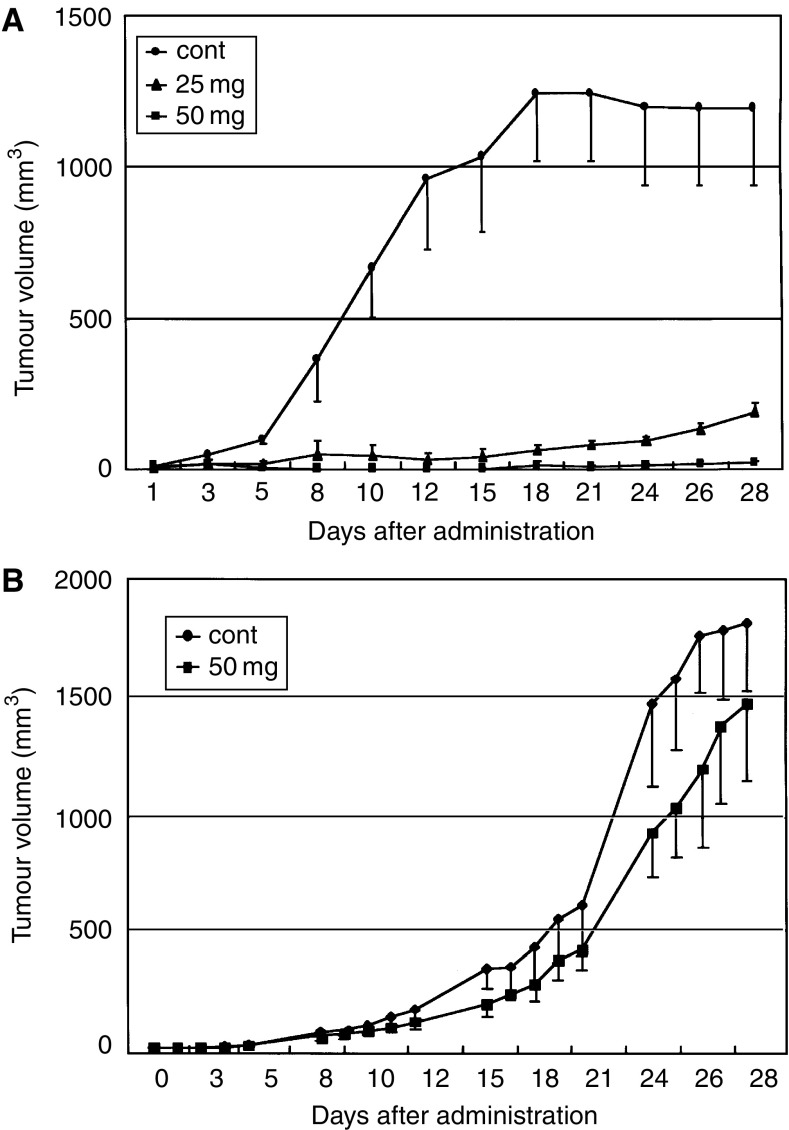
Effects of gefitinib on the growth of xenografts in nude mice was investigated. (**A**) A marked growth inhibition of xenografts of the ACT-1 cell was found in gefitinib-treated mice. Growth of xenografts of the ACT-1 cell was almost completely suppressed by gefitinib at the end of the 4-week treatment period with a dose level of 50 mg kg^−1^. (**B**) No significant growth inhibitory effect of gefitinib was found on the xenografts of the OCUT-2 cell.

**Figure 7:**
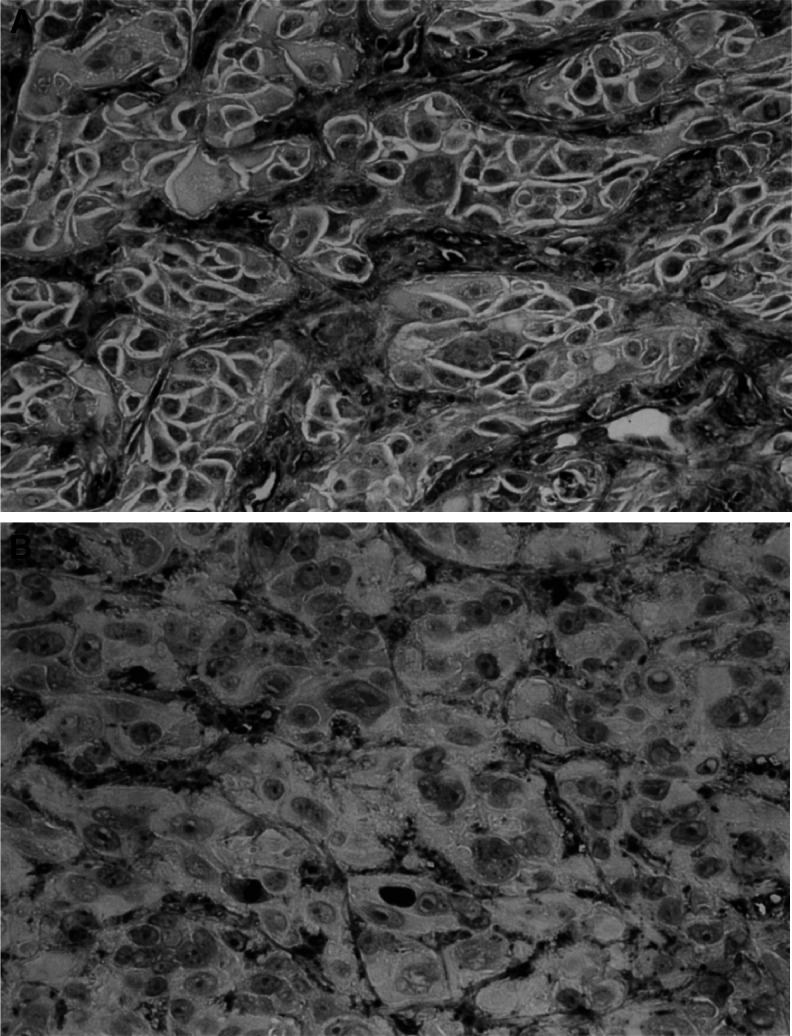
Representative results of immunohistochemistry against CD34 in the xenograft inoculated on the mice. Microvessels in the control group have a thicker wall with wider diameter (**A**) than that of the gefitinib-treated group (**B**). Both images are shown at × 400 magnification originally.

As shown in Figure 6, a marked growth inhibition of xenografts was found in gefitinib-treated mice. This effect was dose dependent. Growth of the xenograft of ACT-1 cells was almost completely suppressed with gefitinib after the 4-week treatment period with a dose of 50 mg kg^−1^. On the other hand, no significant effect of gefitinib administration on the growth of the xenograft of OCUT-2 cells was observed. A reversible mild reduction in body weight was the only side effect observed with the highest dose used. Histological specimens showed marked central necrosis and fibrosis between the cancer cells in xenografts of the ACT-1 cells resected from gefitinib-treated mice though the size was much smaller than that observed in the control group. Immunoreactivity of EGFR on the cells was diminished in xenografts resected from gefitinib-treated mice. CD34-reactive microvessels were universally seen between the viable cancer cells in every xenograft and the density of microvessels did not differ regardless of gefitinib treatment. However, as shown in Figure 7, morphological change of microvessels was demonstrated with gefitinib treatment only in the xenograft of ACT-1. Microvessels in the control group had thicker walls of a wider diameter than those of the gefitinib-treated group. Microvessels in the gefitinib-treated group were scattered between the increased fibrous bands in the xenograft of ACT-1. In contrast, these morphological changes of the tumour vessels were not observed in the xenograft of OCUT-2.

## DISCUSSION

In the present study, we demonstrated that EGFR was almost universally expressed in anaplastic thyroid cancer cell lines, and that the level of its expression varied. Previous studies by immunohistochemistry and EGF receptor assay have also found that EGFR was frequently overexpressed in anaplastic thyroid cancer (Di Carlo *et al*, 1990; Westermark *et al*, 1996). Moreover, in those patients whose thyroid tumours bound more EGF, the prognosis was reported to be poor (Duh *et al*, 1985). These results suggested that EGFR plays a role in determining the malignant potential of the anaplastic thyroid cancer. Recently, EGFR-targeted molecular treatment with tyrosine kinase inhibitors, including gefitinib, has shown significant antitumour activity in clinical trials in advanced NSCLC, with early encouraging clinical data reported in cancer of the head and neck, colon, and breast (Caponigro, 2004). Patients recruited in these trials have failed initial chemotherapy. Therefore, these studies provide a new perspective of anticancer treatment in chemotherapy-resistant tumours, like anaplastic thyroid cancer.

In the present study, however, we could not show significant effects of gefitinib *in vitro* with the normal culture conditions with FBS, initially. In this condition, EGF level in the culture medium was as low as undetectable level (data not shown). Thus, inactivation of the EGFR and its signalling pathway was suggested. As previously stated, the production of many growth factors and cytokines are well known in anaplastic thyroid cancer. The production of these molecules in cancer cells was also suggested to correlate with the highly malignant nature of anaplastic thyroid cancer (Yoshida *et al*, 1994; Ogisawa *et al*, 2002). We thought that there might be a number of activated growth-stimulating pathways, other than the EGF–EGFR pathway, which must contribute to the growth of anaplastic cancer cells in conditions with FBS containing many growth factors. However, in the conditions of activating EGFR with adding EGF in the medium, complete growth inhibition with gefitinib could be demonstrated for EGFR-transmitted signal transduction pathways. Previous preclinical studies have relied on MAPK or Akt activity, representing preserved functional signals from EGFR activation, as a parameter of tumour response to gefitinib treatment (Moasser *et al*, 2001; Moulder *et al*, 2001; Ono *et al*, 2004). Activating mutation in the tyrosine kinase domain of EGFR has also been suggested recently to contribute to the responsiveness of NSCLC (Lynch *et al*, 2004). Still, the mechanism involving the efficacy of gefitinib is not fully understood and controversial results are also reported (Campiglio *et al*, 2004). In the present study, overexpression of EGFR and preservation of its signal transduction pathways, phosphorylation of ERK-1/2 and Akt, were demonstrated in a cell line that responded well to gefitinib. Our results showed that preservation of the EGFR-mediated signal transduction pathway is a necessary condition for EGFR molecular-targeted therapy with gefitinib in anaplastic thyroid cancer cell lines.

Furthermore, gefitinib treatment displayed antitumour activity in tumour-bearing mice. We could demonstrate a dramatic growth suppressive effect of gefitinib on the xenograft of the ACT-1 cells, which highly expressed EGFR, but not on that of the OCUT-2, expressing low level of EGFR. The results were similar to that in ‘*in virto*’ experiment with EGFR activation. These results clearly suggested that the effect of gefitinib on the xenograft was a specific reaction with EGFR on the cells. The efficacy of oral administration of gefitinib is demonstrated much more effectively *in vivo* compared with *in vitro* data. As stated in the results, immunoreactivity of EGFR was diminished in the xenograft of gefitinib-treated mice, suggesting that the gefitinib-resistance potential is low in EGFR-expressing cells. We not only found the effect of gefitinib on the EGFR-overexpressing cancer cells, but also found it on tumour vessels of the xenograft of ACT-1 cells. Growth and metastasis of cancer require several processes; angiogenesis plays a key role (Blood and Zetter, 1990; Hart and Saini, 1992), and it is well known that EGFR signalling pathways have an important role in the regulation of angiogenesis (Bruns *et al*, 2000; Ciardiello *et al*, 2001; Asakuma *et al*, 2004). Hirata *et al* reported that the antiangiogenic effect of gefitinib in the vascular endothelial cells of neovasculature is partly attributable to direct inhibition of EGFR activation, and that endothelial cells in malignant tumours play a critical role in the therapeutic efficacy of gefitinib (Asakuma *et al*, 2004; Hirata *et al*, 2004). We also demonstrated the change in tumour vessels in the xenograft after gefitinib treatment. The inhibition of tumour neovasculature might be one of the reasons that we obtained more significant data from the *in vivo* study. Still, the inhibition of the tumour neovasculature was hardly observed in the xenograft of OCUT-2 cells. Thus, direct effect of gefitinib in the vascular endothelial cells of neovasculature was not enough to explain our results. Expression of vascular endothelial growth factor (VEGF), a strong growth factor for tumour neovasculature, from cancer cells was reported to suppressed after gefitinib treatment (Ciardiello *et al*, 2001). We also found that a significant suppression of VEGF secretion from ACT-1 cells after gefitinib treatment (preliminary data, not shown). Thus, the inhibitory effect of the tumour neovascuature was suggested to be displayed not only a direct but also VEGF-mediated indirect effect of gefinitib, as suggested in previous reports (Hirata *et al*, 2002; Asakuma *et al*, 2004). Further studies should be necessary to clarify the mechanism of the antitumour effect of gefitinib including tumour neovasculature.

Since anaplastic thyroid cancer has a very poor prognosis, a dramatic effect might not be expected from monotherapy with gefitinib in the clinical setting. A combination or a sequential therapy with chemo-, radio- and molecular-targeted-therapy could give a new strategy for highly malignant, chemo- and radio-resistant anaplastic cancer. In conclusion, this study showed the inhibition of EGFR-mediated cell proliferation and the inhibition of tumour angiogenesis by gefitinib in anaplastic thyroid cancer cell lines. This is the first report to describe the potential of EGFR molecular-targeted therapy for treating anaplastic thyroid cancer. Our results suggest that the antitumour effects of gefitinib might offer a new therapeutic approach in anaplastic thyroid carcinoma when functional expression of EGFR is observed.
